# Clinical Benefit of Pazopanib in a Patient with Metastatic Chondrosarcoma: A Case Report and Review of the Literature

**DOI:** 10.3389/fonc.2018.00045

**Published:** 2018-03-01

**Authors:** Onoufrios Tsavaris, Panagiota Economopoulou, Ioannis Kotsantis, Lazaros Reppas, Chrysanthi Avgerinou, Nikolaos Spathas, Maria Prevezanou, Amanda Psyrri

**Affiliations:** ^1^Section of Medical Oncology, Department of Internal Medicine, Faculty of Medicine, National and Kapodistrian University of Athens, Attikon University Hospital, Athens, Greece; ^2^2nd Radiology Department, University General Hospital “ATTIKON”, Medical School, National and Kapodistrian University of Athens, Athens, Greece

**Keywords:** pazopanib, metastatic chondrosarcoma, angiogenesis, vascular endothelial growth factor receptor, platelet-derived growth factor receptor

## Abstract

Chondrosarcoma is a rare malignancy characterized by the production of cartilage matrix, displaying heterogeneous histopathology and clinical behavior. Due to lack of effective treatment for advanced disease, the clinical management of metastatic chondrosarcoma is exceptionally challenging. Chondrosarcomas harbor molecular abnormalities, such as overexpression of platelet-derived growth factor receptor (PDGFR)-alpha and PDGFR-beta, which are required for cancer development, progression, and metastasis. Pazopanib is a potent and selective multitargeted tyrosine kinase inhibitor, which co-inhibits stem cell growth factor receptor (c-KIT), fibroblast growth factor receptor (FGFR), PDGFR, and vascular endothelial growth factor receptor (VEGFR) and has demonstrated clinical activity in patients with advanced previously treated soft tissue sarcoma. Herein, we describe the unique case of a patient with metastatic chondrosarcoma who derived clinical benefit from pazopanib after first-line chemotherapy failure.

## Introduction

Chondrosarcomas are a heterogeneous group of malignant bone tumors that are commonly characterized by the production of chondroid (cartilaginous) matrix (1). The vast majority is conventional chondrosarcomas; rare subtypes include dedifferentiated, clear cell, and mesenchymal chondrosarcomas (2). Histologic grade is the most important indicator of clinical behavior and prognosis. Grade 3 chondrosarcomas are frequently metastatic, and prognosis is poor with surgical resection alone (3). The main metastatic site is the lung, while regional lymph nodes and liver are much less commonly involved (4).

The cornerstone of treatment for localized chondrosarcoma is surgical resection, which offers the only chance for cure. Chemotherapy is considered ineffective; however, it might be effective in the mesenchymal subtype and is of uncertain value in dedifferentiated chondrosarcoma (5). Several signaling pathways, such as hedgehog, non-receptor tyrosine kinase (Src), phosphatidylinositol-4,5-bisphosphate 3-kinase (PI3K)/serine/threonine-protein kinase (AKT)/mammalian target of rapamycin (mTOR), and angiogenesis are suggested to play an important role in chondrosarcoma (6). Among them, angiogenesis is a key biological phenomenon that plays a critical role in tumor formation, invasion and metastasis.

Pazopanib (Votrient, GW786034, GlaxoSmithKline) is a multi-tyrosine kinase inhibitor (TKI) that targets angiogenesis growth factor receptors, such as stem cell growth factor receptor (c-KIT), fibroblast growth factor receptor (FGFR), platelet-derived growth factor receptor, and vascular endothelial growth factor receptor (VEGFR). It has been shown to suppress chondrosarcoma growth in mice (7). Pazopanib has been approved for the treatment of metastatic soft tissue sarcoma after failure of anthracycline-based chemotherapy, based on a pivotal phase III trial, which demonstrated improved progression-free survival compared with placebo (8). Based on these considerations, we have tested the efficacy of standard pazopanib dose of 800 mg daily in a patient with metastatic chondrosarcoma who had disease progression following anthracycline-based chemotherapy.

## Case Presentation

In May 2011, a 41-year-old Caucasian male was referred to the orthopedic department of our institution for evaluation of a 6-month left proximal humeral pain, increasing in severity over the last 2–3 weeks. His medical history was unremarkable and was under no medications at the time. He was an ex smoker and a social drinker and worked in constructions. Family history was notable for a grandfather with lung cancer and an aunt with breast cancer.

Upon physical examination, a limited range of motion of the left shoulder was noted (decreased flexion, extension, abduction, and external rotation). Plain radiograph of the left shoulder demonstrated a lytic lesion with intralesional calcifications and cortical remodeling, suggestive of a chondrosarcoma. MRI of the left shoulder confirmed this finding. Biopsy of the lesion was positive for grade 2–3 chondrosarcoma. A full diagnostic workup was carried out, including a CT of the chest and upper abdomen and a bone scan, which demonstrated increased uptake in the left upper arm but no additional bone lesions. Hematological and biochemical investigations were within normal limits.

In June 2011, the patient underwent a limb sparing surgical operation, and pathology was positive for conventional chondrosarcoma grade 2–3, that had destroyed the medullary and cortical portions of bone reaching into adjacent soft tissue. Distal bony resection margin and skin were both free of tumor infiltration. The patient did not receive any adjuvant therapy.

The patient remained free of disease for 4 years following surgery. In 2015, the patient complained of increasing pain in his left resected arm and shoulder. Imaging confirmed local recurrence. The patient subsequently underwent amputation of his left arm with resection of scapula. Pathology confirmed diagnosis of a grade 2/3 conventional chondrosarcoma. In August 2016, he started to experience significant substernal chest pain with shortness of breath and was admitted to the hospital. A CT scan of the chest revealed multiple pulmonary lesions compatible with metastases. The patient was discussed at the oncology tumor board and it was decided that chemotherapy should be administered. He was treated with chemotherapy combination of doxorubicin 20 mg/m^2^, Ifosfamide 2.5 g/m^2^, and Mesna 2.5 g/m^2^ IV on days 1–3 every 3 weeks. After 3 months of treatment, chemotherapy was discontinued due to disease progression, which included presence of new pulmonary nodules.

**Figure 1:**
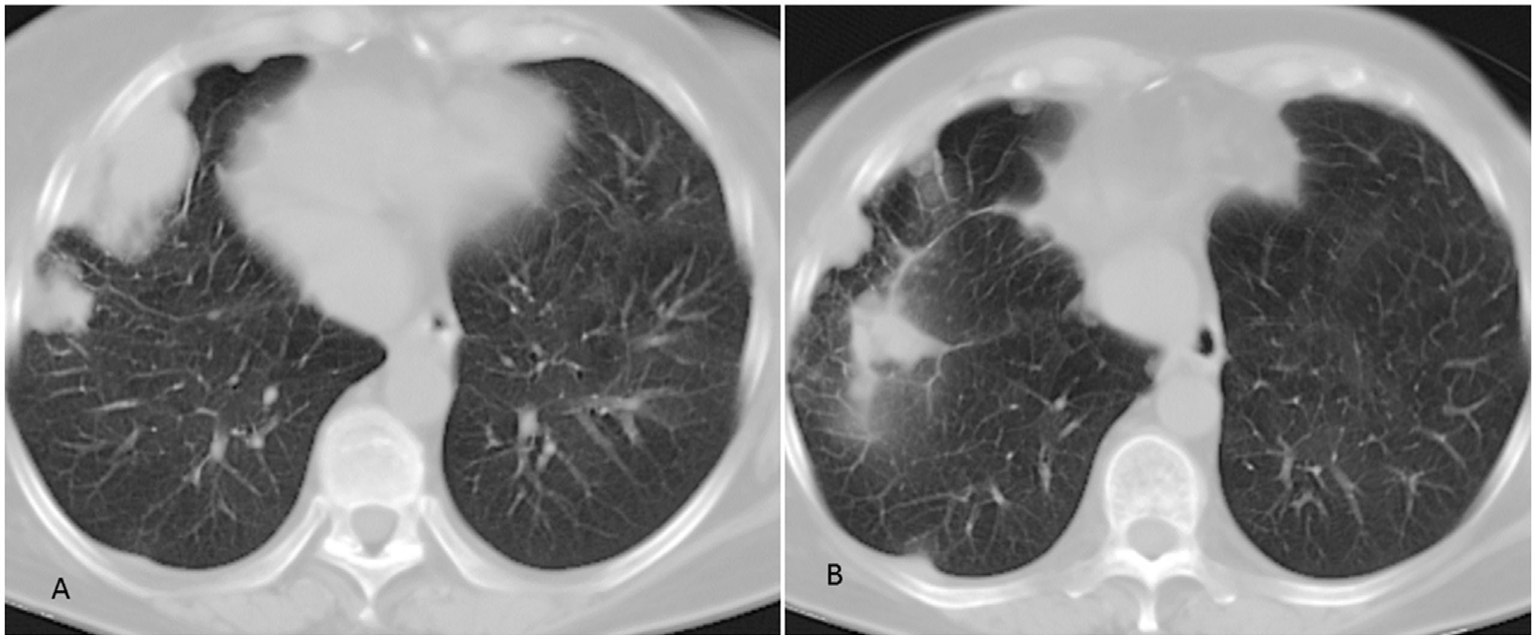
Chest CT scan of the patient. **(A)** Pulmonary lesions of the right lung. **(B)** Partial response of pulmonary lesions following 6 months of treatment with pazopanib.

At that time, we decided to treat the patient with pazopanib as second-line treatment. The drug was well tolerated and after 6 months of treatment, the patient had partial response on imaging according to RECIST criteria (Figure 1), with improvement of symptoms. At the last follow-up in August 2017, clinical, biochemical, and imaging evaluation showed no evidence of disease progression. The patient continues to be on treatment with pazopanib. Written informed consent was obtained from the participant for the publication of this case report.

## Discussion

Due to limited efficacy of available treatment strategies, management of metastatic chondrosarcoma is intriguing. During the last decades, there has been no significant improvement in survival; furthermore, rarity of the disease hampers the conduction of large randomized trials. New therapeutic approaches are urgently needed, and current research focuses on elucidation of molecular events underlying the pathogenesis of this rare malignancy.

Angiogenesis is a hallmark pathway involved in cancer evolution, evasion, and metastasis and has been introduced as an important therapeutic target in the last two decades (9). Preclinical data have supported a role of angiogenesis in the pathogenesis of chondrosarcoma. Early studies have demonstrated production of VEGF in human chondrosarcoma cell lines (10). Chemokine CCL5 has been shown to mediate VEGF angiogenesis *via* inhibition of miR-200b through PI3K/Akt pathway in chondrosarcoma cells (11). In another study, protein hormone adiponectin promoted VEGF-A expression in human chondrosarcoma cell lines *via* PI3k, Akt, mTOR, and hypoxia-inducible factor-1a signaling cascades (12). In chondrosarcoma animal models, treatment with VEGFR2/PFGF-β/FGFR1 inhibitor SU668 resulted in tumor growth inhibition (7). On the other hand, combination of chemotherapeutic agent ET-743 and antiangiogenic protein plasminogen-related protein B has shown to be effective in reducing tumor necrosis in chondrosarcoma animal models (13).

In the case of our patient, angiogenesis TKI pazopanib resulted in objective response for more than 6 months, which was associated with clear improvement of the patient’s symptoms and general condition. Of note, the patient had received prior chemotherapy without any clinical benefit. In a recent report by Jones et al., among eight patients with chondrosarcoma who were treated with pazopanib in the context of a clinical trial, seven patients achieved prolonged disease stabilization for over 6 months (14).

There are two ongoing trials evaluating the efficacy of pazopanib in patients with chondrosarcomas. A phase II study that investigates the efficacy and safety of pazopanib in patients with unresectable or metastatic chondrosarcoma has completed recruitment, and results are eagerly awaited (NCT01330966). In addition, a phase II trial is evaluating pazopanib in patients with unresectable or metastatic solitary fibrous tumor and extraskeletal myxoid chondrosarcoma (NCT02066285). Regorafenib is another oral multikinase inhibitor, which targets angiogenic, stromal, and oncogenic RTKs and is currently being tested in a phase II study in patients with metastatic bone sarcomas (NCT02389244).

## Concluding Remarks

In conclusion, chondrosarcoma is a rare bone malignancy with a diverse pattern of clinical behavior and remarkable resistance to chemotherapy or radiation. Current research focuses on elucidation of molecular events during tumorigenesis, with the ultimate goal to develop effective molecular targeted treatments. Herein, we describe the unusual case of a patient with metastatic chondrosarcoma who derived clinical benefit from pazopanib. Large randomized trials are warranted to confirm a role of pazopanib in chondrosarcoma treatment.

## Author Contributions

OT reviewed the literature and wrote the paper. PE and IK collected the data and contributed in the preparation of the manuscript. CA, NS, and MP contributed in revision of the literature. LR prepared the figure. AP carried out critical interpretations and contributed in the final version of the paper. All the authors read and approved the final manuscript.

## Conflict of Interest Statement

The authors declare that the research was conducted in the absence of any commercial or financial relationships that could be construed as a potential conflict of interest.
